# Lung Abscess Case Series and Review of the Literature

**DOI:** 10.3390/children9071047

**Published:** 2022-07-14

**Authors:** Lamees Yousef, Abdullah Yousef, Abdullah Al-Shamrani

**Affiliations:** 1 Health Holding Company, Vision Realization Office, Khobar 34232, Saudi Arabia; 2 College of Medicine, Imam Abdulrahman Bin Faisal University, Dammam 34211, Saudi Arabia; aaayousef@iau.edu.sa; 3 Department of Pediatrics, Prince Sultan Military Medical City, College of Medicine, AlFaisal University, Riyadh 11533, Saudi Arabia; dr.alshamrani99@gmail.com

**Keywords:** lung abscess, pneumonia, sequestration, bronchogenic cyst

## Abstract

(1) Background: Lung abscess is a lung infection that leads to the destruction of the lung parenchyma, resulting in a cavity formation and central necrosis filled with purulent fluids. It is an uncommon pediatric problem, and there is a paucity of literature reviews on this subject, especially for the pediatric age group. Lung abscess is commonly divided into those considered primary in previously well children or secondary in those with predisposing co-morbidities. The predominant pathogens isolated from primary lung abscesses are the aerobic organisms, including streptococcal species, Staphylococcus aureus, and Klebsiella pneumoniae, while anaerobic bacteria such as Bacteroides species are predominant in secondary groups. Children usually present with fever, cough, shortness of breath, chest pain, and sputum. While physical examination may reveal diffuse crackles on auscultation, the diagnosis is usually confirmed by chest X-ray. (2) Methods: We report four different cases with lung abscesses from both primary and secondary group with similar presentations and radiological findings, but the approach was different in each according to the underlining cause. (3) Conclusions: Conservative therapies with a prolonged course of antibiotics remain the cornerstone of therapy for both primary and secondary lung abscesses. The underlying cause should be considered when there is a suboptimal response. However, invasive intervention is becoming more popular with better yield, shorter duration of antibiotics and admission, and excellent prognosis.

## 1. Introduction

A lung abscess is a circumscribed, thick-walled cavity in the lung that contains purulent material resulting from the suppuration and necrosis of the involved lung parenchyma [1,2]. There are two main types of lung abscesses in children: primary, in which there are no predisposing factors, or secondary, in which there are underlying predisposing factors, either based in the lung or systemic [1]. Other reported classifications include multiloculated and uniloculated, aspirational and hematogenous, or putrid and non-putrid (aerobic and anaerobic) [3]. It can also be classified depending on the duration of the illness into acute, when it lasts 4 weeks or less, or chronic when it lasts longer than 4 weeks [4,5]. Primary lung abscess is predominantly caused by Streptococcus pneumoniae or Staphylococcus aureus [3], whereas secondary lung abscess can be due to anaerobes (27%), Pseudomonas aeruginosa, (13%) Staphylococcus aureus (13%), Streptococcus pneumonia (7%), Haemophilus influenza (7%) [2], Escherichia coli, or klebsiella [4]. Secondary lung abscess is due to several possible structural or functional lung diseases, such as congenital lung malformations, ciliary disorders, cystic fibrosis, immunodeficiencies, aspirations, or infections [1]. Although uncommon, lung abscesses can be further complicated by tuberculosis [6]. The pathophysiology often starts as lung inflammation, followed by necrosis, progressive fibrosis, and cavity formation, leading to the suppurative destruction of lung parenchyma with central cavitation [4]. Lung abscess is a relatively uncommon pediatric illness, with an incidence as low as 0.7/100.000 admissions/year, as reported by Patradoon-Ho and Fitzgerald, and 39 cases/10 years, as reported by Madhani et al. [3]. The morbidity of pediatric lung abscesses was also lower than that in adults [7]. It is rare in neonates and more common in fall and wintertime [8]. Diagnosis is confirmed by chest X-rays, ultrasonography, and computed tomography. Prolonged treatment with empiric broad-spectrum antibiotics is initiated until the causative organism is identified via respiratory culture, which may not be possible in some cases [3]. However, interventional radiology is an alternative that introduces treatment through catheter placement when possible. This treatment approach has the advantage of allowing for quicker recovery from fever and other symptoms, as well as shorter hospitalization times. Other surgical approaches include drainage, aspiration, and resection [5,9]. This report presents three cases of secondary lung abscess and one case of primary lung abscess in children ranging from 13 months to 7 years.

## 2. Case Reports

### 2.1. Case 1

**Figure 1:**
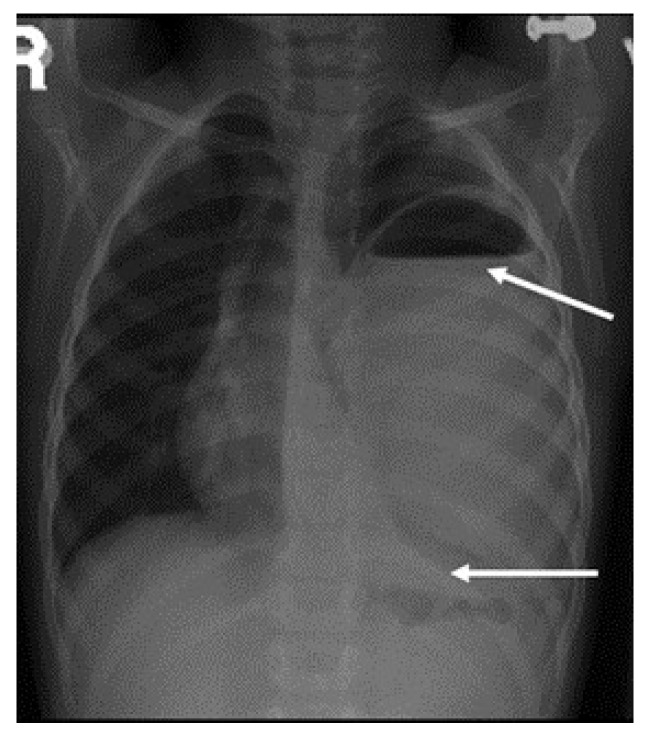
Large left-side cavity with a thick wall filled with thick fluid, with a clear fluid, with a clear fluid level occupying more than 80% of the left-sided lung. The arrows are pointing to the boundaries of the fluid level with mild compression of the mediastinum to the other side and secondary hyperinflation of the right lung.

**Figure 2:**
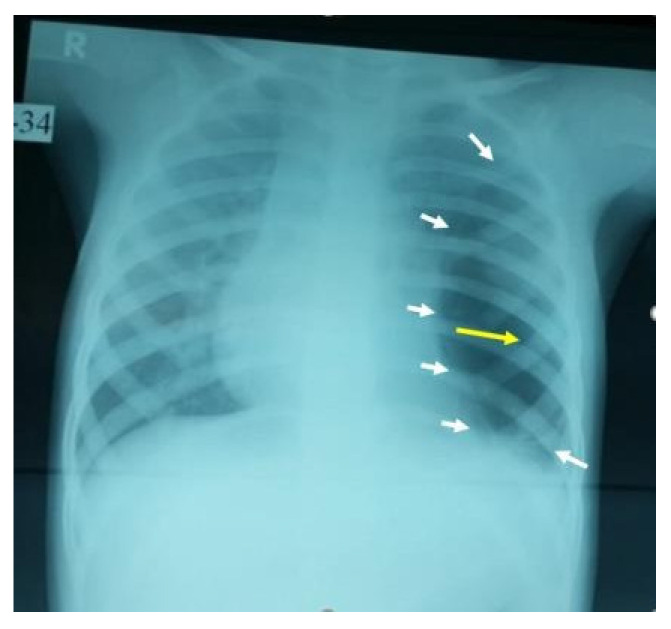
This AP film X-ray shows a large cystic cavity with a marked attenuated vascular marking on the left middle and lower zone, with a clear compression on the heart and ipsilateral hemidiaphragm, raising the possibility of congenital cavity lung lesion, especially CPAM-1 (the white arrows show the borders of the cyst, while the yellow arrow points to the center of the cavitation).

A 3-year-old girl was referred from Najran hospital for further evaluation of complicated pneumonia. She was fully vaccinated, thriving, and had had a recent chest infection several weeks back. She presented with fever, and shortness of breath for a week. On physical exam, she was ill and febrile, with markedly reduced breath sounds on the left side. She had no clubbing as well as no organomegaly but had a good BCG scar. On admission, the chest X-ray showed a large left-sided lung abscess (Figure 1). Previous chest films were retrieved and showed hyperlucency and a large cavity on the left side of the lung, suggesting congenital pulmonary airway malformation (CPAM)-1 (Figure 2). Blood work showed marked leukocytosis with a left-sided shift and very high acute-phase reactant (CRP, ESR), along with negative blood and sputum cultures. The chest CT confirmed the diagnosis of lung abscess and CPAM-1 with no evidence of feeding vessels to the left lower lobe. Lymphocyte subset analysis was not requested, as we believed the primary cause was related to lung malformation. As the patient was initially sick with a large lung abscess, she was managed as a patient with a lung abscess secondary to CPAM-1 and received 4 weeks of IV therapies (vancomycin and meropenem) based on the infectious disease team’s recommendation, which matched our local guideline. She was then scheduled for lobectomy of the left lower lobe. The patient had a smooth course of recovery with no reported complications and completed 8 weeks of antibiotic therapy.

### 2.2. Case 2

**Figure 3:**
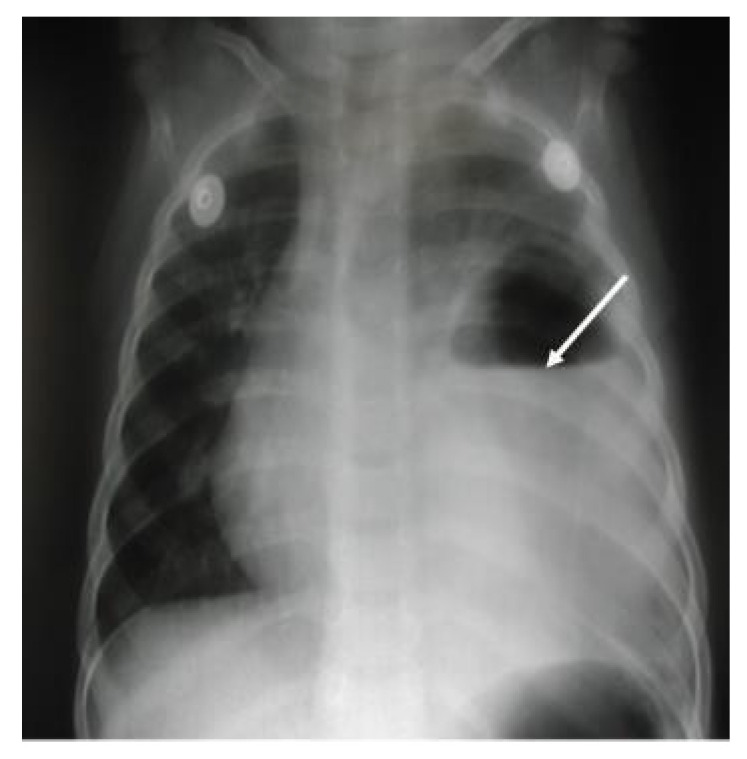
Chest X-ray, AP film, large homogenous density occupying most of the side of the hemithorax with a large amount of air fluid in the superior aspect, which was consistent with a large lung abscess. There was still some shift of the mediastinum to the right, with mild hyperinflation on the right side of the lung.

**Figure 4:**
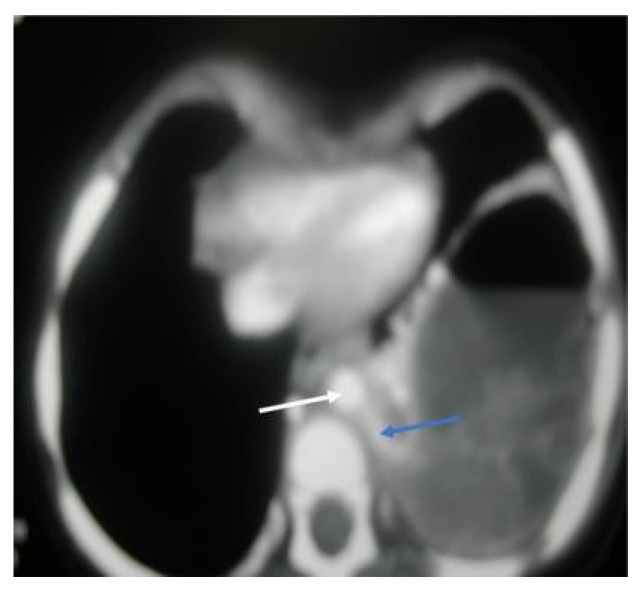
CT chest, axial window, mediastinal view: large, thick wall cavity filled with air fluid with heterogenous opacity. The white arrow shows a descending aorta, while the blue arrow confirms a large feeding vessel to the left lower lobe, a rising and descending aorta, mild compression of left upper lobe, and a mild pectus excavatum.

A 13-month-old girl with complicated pneumonia was referred from a secondary hospital for further evaluation. She was a previously well child with an unremarkable history. She had a flu-like illness and then spiked a high-grade temperature associated with a wet cough and respiratory distress for 3 days. The patient’s condition was diagnosed initially as pneumonia and treated with augmenting for 3 days. Due to inadequate improvement, she was referred to a tertiary center for further management. On physical examination, the patient was ill, with the following vital signs: temperature, 38.7 °C; HR, 130/m; respiratory rate, 60/m; saturation, 88% in room air with a decreased breath sound on the left side. Investigations showed WBC:7.3 k/µL, band: 1.3 k/µL, Hb: 10.4 g/dl, and platelets: 519 k/µL. The chest X-ray suggested complicated pneumonia with a potential lung abscess. She was admitted to intensive care and was started on clindamycin and cefotaxime. A chest tube was inserted on the left side, and 50 mL of pus was drained. The patient’s culture was positive for non-typable Haemophilus influenza, which was sensitive to ceftriaxone and cefuroxime. On the 7th day, the patient was afebrile; she improved and was weaned off oxygen, the chest tube was removed, and she was transferred to the general ward for IV antibiotics. On the 14th day of admission, the patient remained afebrile, with better aeration on the left side, and had reassuring blood works. Therefore, she was discharged on cefuroxime for two more weeks with a follow-up in OPD. Unfortunately, 5 days later, the patient was readmitted with a high-grade fever, wet cough, shortness of breath, and grunting with a marked decrease in breath sounds on the left side. Otherwise, the systemic examination was normal, and compliance with antibiotics was excellent. The repeated chest X-ray on the second admission was similar to the initial presentation (Figure 3). The diagnosis was consistent with a left-sided lung abscess. Immune workup was normal, and computed tomography of the chest with a contrast confirming lung sequestration was performed (Figure 4). The final diagnosis was a left-sided lung abscess secondary to pulmonary sequestration of the left lower lobe. The patient was treated with an IV antibiotic for several weeks until the fever was controlled and the acute phase markers improved. The treatment proceeded for the lobectomy of the left lower lobe with the ligation of the blood vessel. The patient had a smooth course of surgery with no reported complications.

### 2.3. Case 3

**Figure 5:**
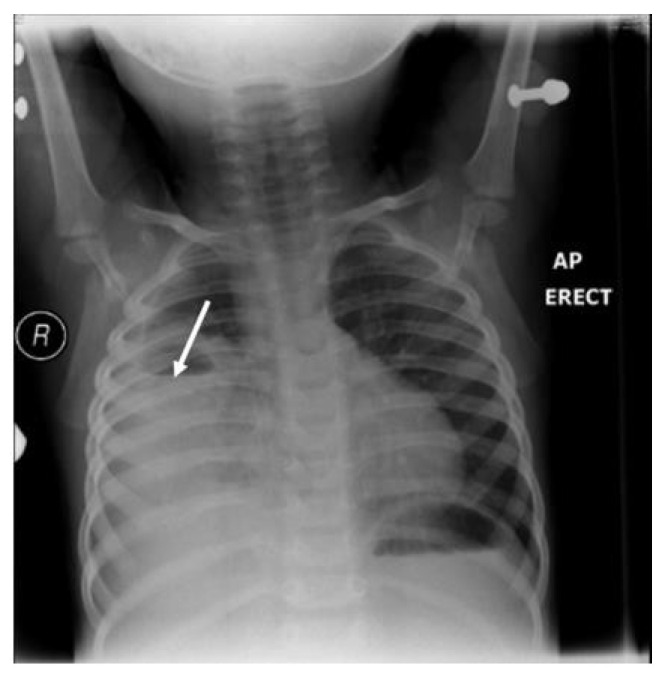
Large homogenous added density occupying most of the right side of the hemithorax with a large amount of air fluid in the superior aspect, as shown by the white arrow, silhouetting the cardiac border, costo- and cardiophrenic angles with no air bronchogram, suggesting large right-sided lung abscess with secondary mild hyperinflation on the left side of the lung.

A 21-month-old boy presented with an acute history of fever, cough, diarrhea, and poor appetite for a week. His medical history was unremarkable. On examination, he looked unwell, and his vital signs were as follows: temperature 38.9 °C, heart rate 160/m, respiratory rate 36/m, blood pressure 93/56 mmHg, and oxygen saturation 95% in room air. There was no finger clubbing. Chest examination revealed tachypnea and the use of accessory muscles (sub-costal and intercostal muscles) with markedly reduced breath sound on the right side, no bronchial breathing, a mild tenderness of the right hypochondrium, no lymphadenopathy, and the rest of the systemic exam was normal. Investigations had the following results: white blood 17.2 k/µL, predominantly neutrophils 12.4 k/µL, while lymphocytes 3.6 k/µL, Hb 9.8 g/dl, platelets 567 k/µL, C-reactive protein 157 mg/L, erythrocyte sedimentation rate 70 mm/h, normal electrolytes, negative blood culture, and negative mono spot test. Chest X-ray suggested a right-sided lung abscess (Figure 5), CT chest confirmed a large lung abscess and sequestration of the right lower lobe. The patient was treated with cefotaxime and vancomycin for 3 weeks. The patient was then scheduled for surgery (resection of the right lower lobe). He then completed 6 weeks of total therapy with no reported complications.

### 2.4. Case 4

A 7-year-old child was brought to the emergency department with an acute history of fever, cough, and chest pain for 3 days. She was previously well, had no history of any medical illness, and was fully vaccinated and thriving. She had no family history of any chronic illness.

**Figure 6:**
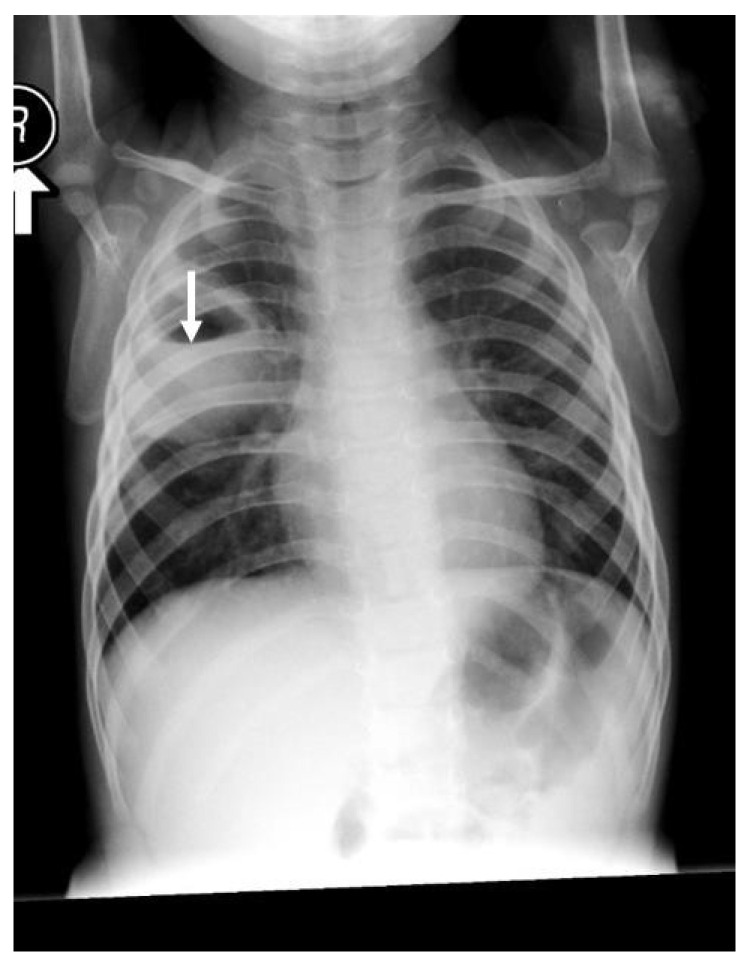
Large, homogenous, rounded, density of 3 × 3 cm in the right upper zone with a large amount of air fluid and thick wall cavity. The white arrow shows the upper part of the fluid level.

On examination, the patient looked ill and febrile at 39 °C. Her respiratory rate was 35/m, the room air saturation was 97%, her heart rate was 130/m, her blood pressure was 106/70 mmHg, and her fingers were not clubbed. She had asymmetrical breath sounds, decreased breath sound on the right side, and little pain on inhalation, with no wheeze or crackles. The investigations had the following results: WBC was 20 k/µL, neutrophils were predominant, platelets were 500 k/µL, Hb was 10.9 g/dl, and sputum culture was positive for Staphylococcus aureus. The chest X-ray showed a small, right-sided lung abscess (Figure 6). She was treated as a case of primary lung abscess with ceftriaxone and clindamycin. On the fourth day, the chest CT showed resolution of fluid. She was treated for 3 weeks, and the follow-up showed complete recovery with the disappearance of the cavity.

## 3. Discussion

The literature on pediatric lung abscess case series is scarce [3]. Dhanushke et al. reported unwarranted variation in the management of pediatric lung abscesses with an alarmingly prolonged hospitalization [10]. Studies have reported fever, cough, and dyspnea as the most common symptoms of lung abscess [11,12].

In our study, the most common symptoms were fever (100%), cough (75%), chest/abdominal pain (50%), and dyspnea (50%), with a mean duration of 13 days (range 3–56). This matches what has been reported in the literature [3,11,12]. The patients in our cases had a dry, non-productive cough initially, but when communication with the airway happened, the cough changed to be wet and productive [11,12]; none were clubbed, which could be seen in severe cases. 

Lung abscesses can develop in any part of the lung [11], and the most common site of the abscess varies between reported studies. Two of our cases had left-sided lung abscesses, while the other two had right-sided lung abscesses, and 50% of our cases were in the left lower lobe, similar to Madhani et al., 33% [3].

The differential diagnosis may include congenital lung cyst, hydatid cyst, and mycobacterium [5].

The organism was identified in two cases, 50% (the second case from the aspirated fluid and the fourth case from the sputum culture). Despite the appropriate antibiotic based on sensitivity, the second patient progressed to surgical intervention due to the presence of sequestration as a predisposing factor. The yield of blood cultures in our cases was negative. Currently, the increase in the interventional approach might increase the yield of pathogens responsible for lung abscess from 30% to potentially 60% [10,13,14].

Chest radiography is often the diagnostic method of choice as in our cases [3]; however, chest CT is often indicated in suboptimal response prior to intervention, differentiating lung abscess from empyema and ruling out potential lung malformation [12,14].

Yunus found that 42% of 19 patients with lung abscesses encountered no complication in CT guidance catheter placement. The most common complication was pneumothorax (26%). Luckily, no complication was reported in our cases [15].

Bronchoscopy and lavage could be utilized if the patient is still symptomatic after the first week of conservative therapy to retrieve organisms and exclude endobronchial obstruction [5]. Contributing factors to lung abscess in children should be looked at carefully, including lung malformation such as as congenital cyst, gastroesophageal reflux disease, immunodeficiency, weak cough, and immunosuppressive agents [5,9,12]. Three of our cases were secondary lung abscesses, and only one was primary. However, in a newly published Australian study, it was found that among 68 cases, 81% were predominantly primary and Staphylococcus aureus (including MRSA) was the commonest organism at 80% [10].

There are several approaches to treating lung abscesses, ranging from conservative approaches such as standard therapy to different types of surgical interventions [1,3,5,9,12].

The standard course of treatment involves an extended period of intravenous antibiotics for 24 days [3]. However, Chan et al. found the mean duration of IV antibiotic treatment was 40 days [9]. Madhani et al. reported patients who received IV antibiotics to be 28 days for primary lung abscess and 45 days for secondary lung abscess. Third-generation cephalosporin (cefotaxime or ceftriaxone) and clindamycin were the initial therapies in 75% of our cases, similar to what has been recommended by Madhani et al. [3]. The duration of therapies in our cases ranged from 3 weeks, as in the fourth case, to 8 weeks, as in the first one. Combination therapies can be tailored according to the response, radiological change, and acute phase reactants result as in the first case in which a more potent antibiotic commenced [11,16].

Treatment with IV antibiotics can fail if the abscess is larger than 6 cm or if the patient has other contributing factors [17]. The next treatment option is surgical intervention, which ranges from chest tube insertion, pigtail catheter placement, video-assisted thoracoscopy, or lobectomy [5,14].

Pigtail catheter has shown a quicker recovery time from fever and other symptoms, as well as less overall hospitalization for lung abscess [5,9].

The three secondary lung abscesses in our study were treated with IV antibiotics for a prolonged period, after which a lobectomy was performed, whereas the primary lung abscess case was managed with IV antibiotics only and had a full recovery. As the literature suggests, pediatric patients who have complicated cases of lung abscess or do not show clinical or radiological improvements in their condition after being on the recommended course of IV antibiotics or after having simple drainage performed might need surgical procedures such as lobectomy [11].

Similarly, Wali et al. argued that although 80–90% of patients with lung abscesses can be successfully treated with antibiotics, this treatment can also fail. If that happens, pulmonary resection is the advised course of treatment [13].

In our study, the first case was referred to the tertiary hospital for further evaluation for complicated pneumonia. Her left-sided lung abscess was confirmed via CT scans, and she was managed non-surgically through IV antibiotics of vancomycin and meropenem for a period of 4 weeks. After that, the patient received a lobectomy of the left lower lobe and an additional 4 weeks of antibiotics. Similarly, the second case was also a complicated case of pneumonia and was referred to the hospital for further evaluation. The patient had a wet cough and a high-grade, persistent fever. The CT scans revealed complicated pneumonia with a suspected lung abscess.

The patient was started on IV antibiotics of clindamycin and cefotaxime, and drainage was performed. She completed 14 days of IV antibiotics and was slightly improving before returning 5 days later with a worsening condition. The patient was diagnosed with a left-sided lung abscess secondary to pulmonary sequestration and was put on further antibiotics until she improved. She was then treated with lobectomy. The third case was a 21-month-old boy with a fever and cough, and reduced breath sounds on the right side but an otherwise normal exam. Blood culture was negative, and CT scans showed a right lower lobe lung abscess and sequestrated right lower lobe. The patient was treated with cefotaxime and vancomycin for 3 weeks and scheduled back for a lobectomy after 6 weeks of IV antibiotic treatment due to the large size of her lung abscess and sequestration. The only case in this study that was managed by antibiotics alone was the only patient with a primary lung abscess. This patient, who was previously well, had an acute history of fever, cough, and chest pain for 3 days. The sputum culture was positive for Staphylococcus aureus, and the chest X-ray showed a small right-sided lung abscess. The patient received ceftriaxone and clindamycin, and by the fourth day, CT scans already showed resolution of the fluid. The patient received a total of three weeks of treatment with complete resolution of lung cavitation on chest X-ray.

Based on our surgical cases, the interventions’ duration varied (four weeks, one week, and six weeks) with a mean of 3.7 weeks which is approaching the definition of chronic illness, which lasts more than 4 weeks [5].

In a retrospective review of cases in the literature, Yen et al. reported that 39% of their patients had been treated non-operatively by IV antibiotics solely or together with a percutaneous drainage procedure [11]. While surgical intervention is not always the selected approach [3], the remaining majority, i.e., 61%, had to be managed surgically [11]. Madhani et al. found that only 10 cases out of 39 had received surgical intervention [3]. Based on the presented cases and the data available in the literature, surgical treatment for lung abscess might be needed when a lung abscess patient does not respond to intravenous antibiotic treatment alone [11,16,18]. Surgical drainage under CT guidance with a pigtail catheter is very beneficial when the response is unsatisfactory [5]. Video-assisted thoracoscopy is gaining popularity, whereas lobectomy is rare nowadays [5,9].

## 4. Conclusions

Appropriate IV antibiotic therapy alone is recommended as initial therapy for primary or secondary lung abscesses in children. If the initial treatment failed or was suboptimal, then the percutaneous aspirate is advisable before proceeding to more invasive procedures.

Surgical drainage with a pigtail catheter is a minimally invasive procedure and the procedure of choice prior to proceeding to more invasive intervention. Pediatricians should be alerted to the potential underlying cause, especially if there was no optimal response to the initial therapies. The prognosis of lung abscesses is often excellent.

## Data Availability

Further information regarding the cases could be obtained by contacting the corresponding author.
